# Childhood Cancer Data Initiative Participant Index: Mapping Pediatric Cancer Data to Facilitate Cross-Study Integrated Analysis

**DOI:** 10.1200/CCI-25-00387

**Published:** 2026-04-08

**Authors:** Subhashini Jagu, Jaime M. Guidry Auvil, Mark D. Cunningham, Ricardo Flores Jimenez, Martin L. Ferguson, Malcolm A. Smith, Douglas S. Hawkins, Todd A. Alonzo, Thalia Beeles, Brigitte C. Widemann, Warren A. Kibbe, Gregory H. Reaman

**Affiliations:** ^1^ Office of Data Sharing, National Cancer Institute, Bethesda, MD; ^2^ Frederick National Laboratory for Cancer Research, Leidos Biomedical Research, Inc, Frederick, MD; ^3^ Essential Software Inc, Gaithersburg, MD; ^4^ Clinical Investigations Branch, National Cancer Institute, Bethesda, MD; ^5^ Children's Oncology Group, Monrovia, CA; ^6^ Department of Preventative Medicine, University of Southern California, Los Angeles, CA; ^7^ Pediatric Oncology Branch/Center for Cancer Research, National Cancer Institute, Bethesda, MD; ^8^ Data Science and Strategy, National Cancer Institute, Bethesda, MD; ^9^ Division of Cancer Treatment and Diagnosis, National Cancer Institute, Bethesda, MD

## Abstract

**PURPOSE:**

To facilitate integrated multimodal data analysis, it is critical to connect data from multiple sources to address multifaceted research questions, better understand disease biology and natural history, develop new therapies, and improve existing treatments. The Childhood Cancer Data Initiative (CCDI) Participant Index, an application programming interface, aims to address this challenge by providing a digital ID mapping and matching service which collects and cross-references all known IDs associated with a participant.

**METHODS:**

A variety of retrospective and prospective data collected through the CCDI Data Ecosystem equal or surpass the complexity of patient data systems in large health care organizations. The CCDI Data Ecosystem includes participant data collected under multiple protocols and at multiple sites, which often results in the same participant being associated with multiple IDs depending on the source, time, or other variables. CCDI is exploring ways to integrate diverse data types (such as genomic, proteomic, imaging, transcriptomic, clinical trial, and electronic health record data), collected over time and from different sources at the participant level, while ensuring privacy protection.

**RESULTS:**

This mapping allows researchers to access a more complete picture of a participant, even when data are collected at different time points, organizations, protocols, and consents.

**CONCLUSION:**

This facilitates the creation of a connected data ecosystem and promotes data reuse, which, in turn, can accelerate research and improve participant outcomes.

## INTRODUCTION

The goal of the Childhood Cancer Data Initiative (CCDI)^1^ is to harness the power of data and community to transform childhood cancer research. The CCDI Data Ecosystem (CCDE)^2^ aims to gather and integrate data from every child and adolescent and young adult (AYA) diagnosed with cancer and to develop tools that connect preclinical, clinical, research, real-world, and population data.^3-6^

Currently, various childhood cancer data types are generated under different protocols, across diverse studies and projects, at numerous institutions, in different laboratories within an institution, and at different time points. These data are stored in separate repositories and labeled with participant research identifiers (IDs) that are unique to each study/project. As a result, the ability to learn from childhood cancer data is limited because a participant's information becomes fragmented across studies and systems. To enable integrated data analysis, it is crucial to cross-reference a participant's data across multiple sources.^7^

Any childhood and AYA cancer participant may contribute to several independent studies, resulting in multiple IDs and data distributed across repositories such as the database of Genotypes and Phenotypes (dbGaP),^8^ AACR Project GENIE,^9^ Children's Oncology Group (COG),^10^ Pediatric Cancer Data Commons,^11^ Childhood Cancer Clinical Data Commons (C3DC),^12^ and National Childhood Cancer Registry (NCCR);^13^ programs like the National Cancer Institute's (NCI's) Therapeutically Applicable Research to Generate Effective Treatments (TARGET); and at hospitals, local academic medical centers, and other locations. Many participants are also enrolled in clinical trials, with data ultimately deposited into repositories like the National Clinical Trials Network (NCTN) Data Archive^14^ and other platforms. In addition, biospecimens may be used in derivative studies, such as the development of cancer organoids, patient-derived cell lines (PDCLs), or patient-derived xenograft (PDX) models, each of which may generate new IDs that are not often mapped back to the original donor of the tissue. To address multifaceted research questions, understand disease biology, develop new therapies, and advance existing treatments, it is critical to connect data from these multiple sources.^15^

The CCDI Participant Index (CPI) addresses this challenge by serving as a central registry that cross-references project-specific participant IDs to ensure that data from the same individual can be mapped across studies and resources.^12^

### CCDI Data Ecosystem

The CCDE is a network of interconnected data and tools that brings together disconnected clinical, research, and cancer registry data on children and AYAs. It enables researchers' ability to access information needed to address critical scientific questions by streamlining data collection and harmonizing data to common standards.

The CCDI Hub serves as the central data portal and a unified access point to the CCDE, allowing users to explore, search, and connect pediatric cancer data, tools, and resources. A key objective of CCDI is to maximize the use of data by linking information across resources. The CCDI Hub Explore Dashboard^16^ brings together new and existing data from multiple platforms and indexes it at the file level. It serves as a primary reference point for finding data associated with a participant across resources. Within the Hub Explore Dashboard, participant research identifiers and mapped IDs are displayed, which are essential for connecting data across repositories and registries.

### Connecting Data Through the CPI

**FIG 1.**
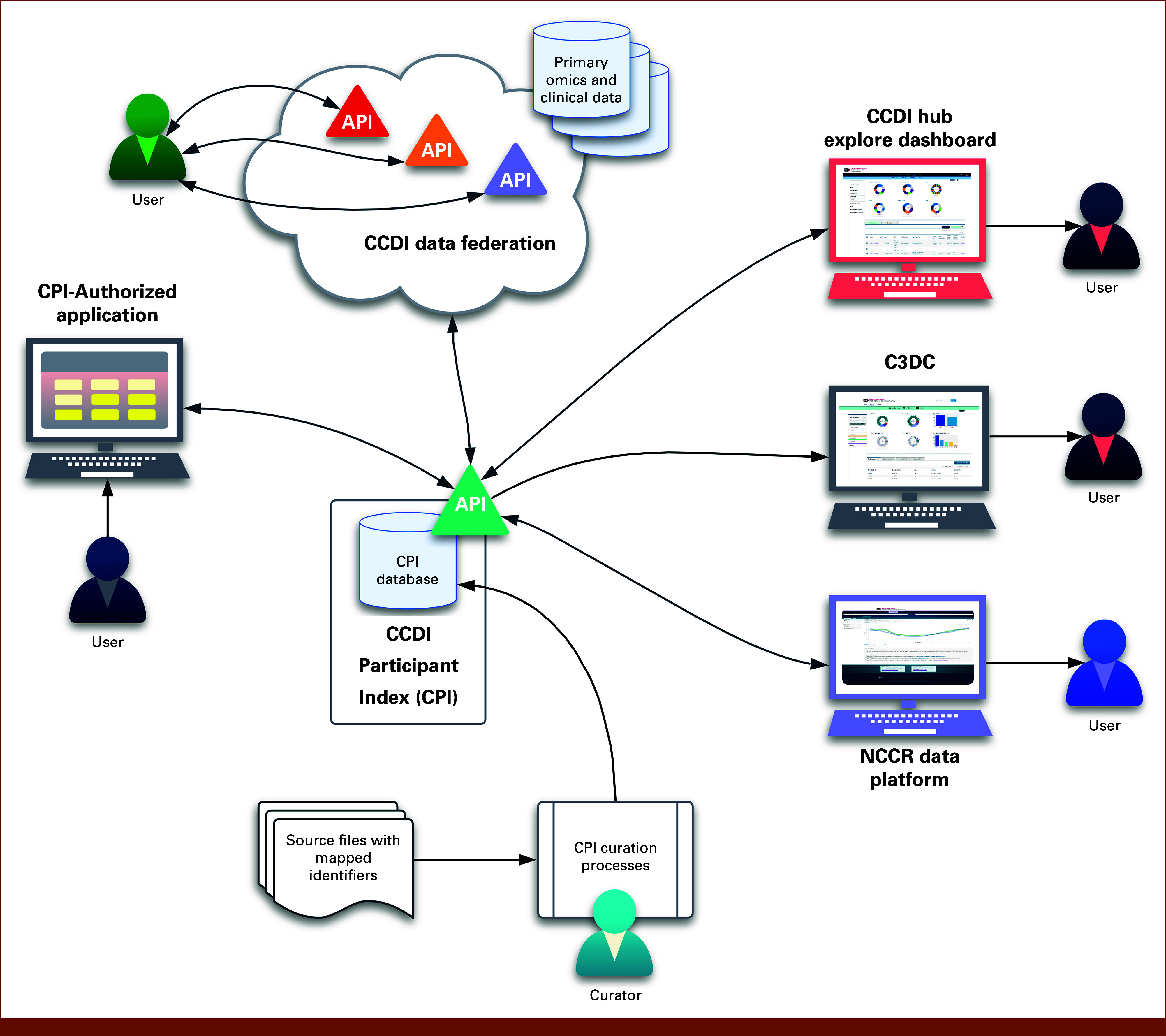
Integration of data across the CCDI ecosystem through the CPI. This diagram illustrates how data are connected through mapped identifiers collected from multiple sources to enable cross-study linkage. The CPI supports downstream access for authorized applications and distributes mapped identifiers to core CCDI resources, including the CCDI Hub Explore Dashboard, C3DC, the NCCR Data Platform, and CCDI Data Federation. API, application programming interface; C3DC, Childhood Cancer Clinical Data Commons; CCDI, Childhood Cancer Data Initiative; CPI, CCDI Participant Index; NCCR, National Childhood Cancer Registry.

The CPI collects and cross-references project- or study-specific participant IDs that represent the same individual across data sets and repositories,^12^ thereby supporting the integration of multimodal data needed to address complex research questions. It is an online database that functions as a backend reference service for the CCDE and acts as a central interoperability layer, linking participant-level data across CCDI resources such as the CCDI Hub Explore Dashboard, C3DC, and the NCCR. The CPI also supports integration across the CCDI Data Federation,^17^ which includes the Gabriella Miller Kids First Data Resource Program (Kids First),^18^ St Jude Cloud,^19^ the UCSC Treehouse Childhood Cancer Initiative (UCSC Treehouse),^20^ PCDC,^11^ the Childhood Cancer Catalog of Circular Extrachromosomal DNA,^21^ the Pediatric Solid Tumor Program at the Indiana University Simon Comprehensive Cancer Center, and other entities such as COG,^10^ dbGaP,^8^ and the AACR Project GENIE^9^ (Fig 1).

The associations between these IDs are gathered and curated from a variety of sources, including research data submissions that include references to participants' identifiers in other studies, mapped identifiers provided by research institutions, and dbGaP's Subject Sample Telemetry Report (SSTR).

The CPI database maintains mappings between identifiers, but it does not store any underlying data. It provides publicly shareable, nonpersonally identifiable information (PII) research IDs through its application programming interface (API) to enable secure and compliant linkage across resources. Importantly, retrieval of any primary data remains under the control of the source repository and occurs independent of the CPI.

### Integrating COG Identifiers Into the CPI

The COG, supported by NCI, is the world's largest organization focused on pediatric and AYA cancers. It includes more than 200 institutions in the United States, Canada, Australia, and New Zealand that provide care for more than 80% of children with cancer in North America and conduct clinical trials, as well as epidemiology and correlative biology studies, in multiple different pediatric cancers.^13^

Each participant enrolled in a COG clinical trial or study is assigned a COG Registration Identifier (COG ID). Every participant with a COG ID is also assigned a COG Universal Specimen Identifier (COG USI), regardless of whether they have submitted a specimen. Each COG patient retains the same COG ID and COG USI throughout their participation, even if they enroll in multiple COG studies. To preserve the organization's honest broker role, these two identifiers are intentionally maintained separately.^7^

Given that the COG ID is the most widely assigned participant identifier in childhood cancer in North America, it serves as a powerful anchor point in the CPI to cross-reference research IDs and integrate data sets. To support data integration and interoperability, COG is collaborating with CCDI by sharing its honest broker status to populate the CPI with a regularly updated list of COG ID-USI pairs. The CPI safeguards COG IDs to maintain compliance under COG's honest broker model. This framework allows linkage with other shareable research identifiers and previously disconnected data sets, thereby greatly enhancing research value.

### Use Cases and Impact

The following use cases demonstrate how the CPI enhances data connectivity across preclinical, clinical, real-world, registry, genomic, and imaging data sets and how linking research or deidentified identifiers can unlock opportunities for timely and richer analyses, reduce duplication of effort, and accelerate discoveries in pediatric cancer research.

*1. Linking COG eligibility screening protocol and clinical trial data:* Linking COG's screening protocol pretreatment and diagnostic data with later-phase clinical trials using COG USIs enables longitudinal, participant-level views of therapy and outcomes across multiple study networks.
Examples: Mapping of CCDI's Molecular Characterization Initiative (MCI)^6^ clinical testing data with institutional treatment data; integrating telomere maintenance data from COG's phase III studies with CCDI's MCI clinical testing data; or overlaying MCI mutational data with pesticide exposure data from the US Geological Survey.
*2. Integrating radiation oncology/imaging data:* Integrating radiation oncology and imaging data collected at external sites with COG clinical data supports joint analyses and improves cross-institutional data sharing.
Examples: Connecting COG neuroblastoma data with the New Approaches to Neuroblastoma Therapy consortium data; mapping imaging data in The Cancer Imaging Archive with clinical trial data in the NCTN Data Archive and the genomics data in the CCDE.
*3. Linking patient-derived models and molecular data:* Mapping PDCL/PDX small-molecule testing data that are often stored separately from the corresponding participant's clinical and omic information accelerates the translational bridge between preclinical research and clinical outcomes and supports drug discovery.
Example: Linking RNA sequencing and drug response profiles from PDCLs or PDXs with participant's MCI clinical testing data.
*4. Connecting electronic health record (EHR) treatment data with research data sets:* Integrating deidentified EHR treatment data with data in the CCDE improves completeness and interoperability of treatment information across institutions.
Example: Mapping treatment and clinical outcome data from Children's Healthcare of Atlanta and the Children's Hospital of Philadelphia with genomics data from the Children's Brain Tumor Network in the CCDI Hub Explore Dashboard.
*5. Linking population-based registry and multimodal research data:* Mapping population-based registry data (phenotypic and clinical) and registry-linked data sets from the NCCR Data Platform to multimodal data in the CCDI Hub Explore Dashboard enables researchers to assess clinical trial participation, treatment outcomes, survivorship, and late effects across subpopulations.
Example: Using linked registry data and multimodal research data sets to identify patterns in long-term health outcomes among pediatric and AYA cancer cohorts.

### System Description

The CPI stores mapped IDs, but data access remains under the control of the primary resource. The CPI system functions can be divided into two broad and operationally separate activities:

• ID registration, ingestion, and management
• ID querying and retrieval.

### ID Registration, Ingestion, and Management

**FIG 2.**
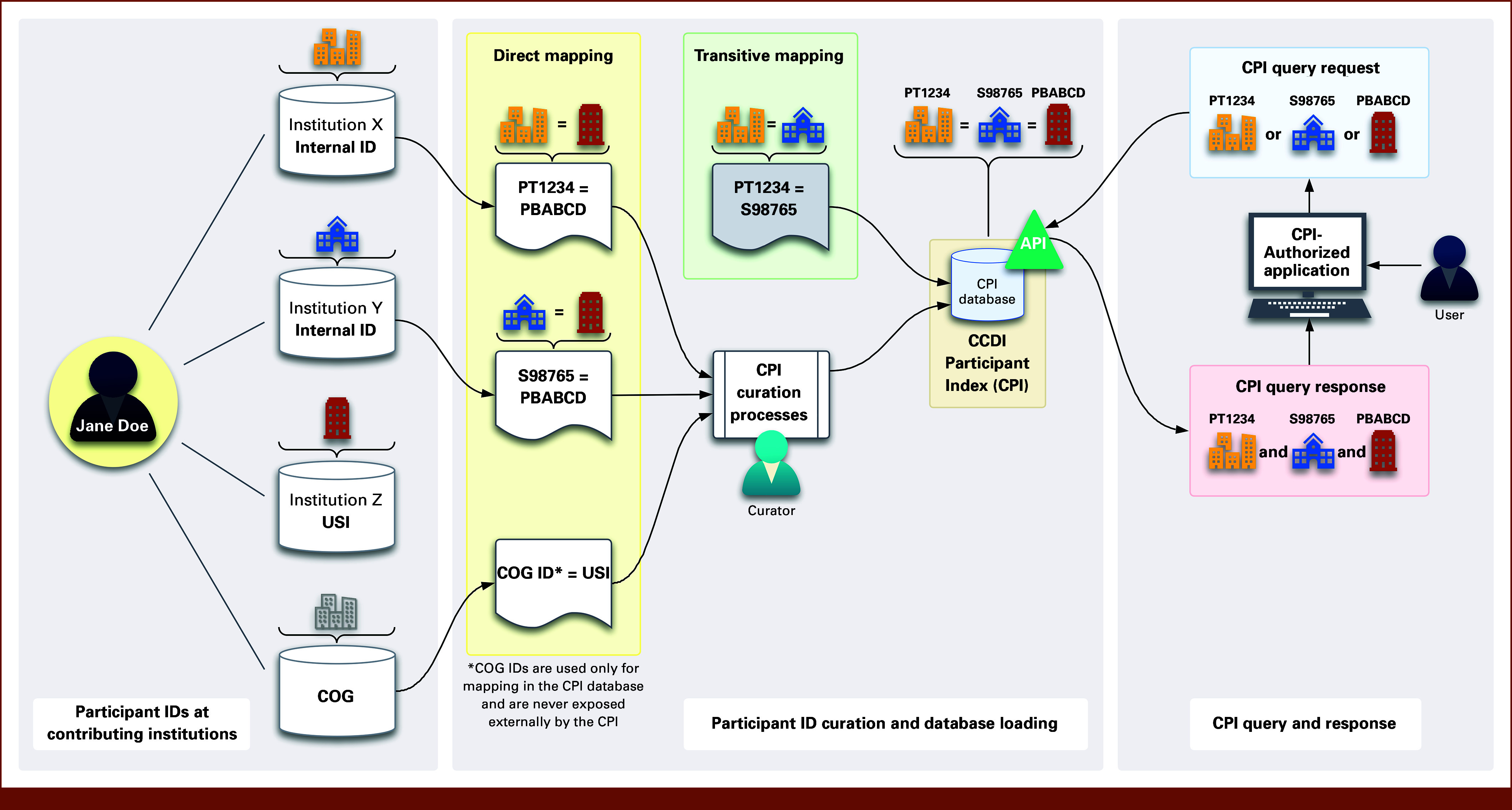
Overview of participant identifier mapping, query, and retrieval processes in the CPI. This diagram illustrates how participant identifiers from multiple contributing institutions are mapped, curated, and retrieved within the CPI. On the left, all known identifiers associated with the same participant are collected from data submitters. The CPI curation process establishes cross–data set linkages using these submitted IDs, identifying those that are likely to represent the same participant through direct and transitive mapping. The curated mappings are stored in the CPI database, where they can be accessed through secure APIs (middle). On the right, authorized users can submit CPI query requests via approved applications to retrieve linked participant identifiers. The system returns mapped IDs across institutions and studies, facilitating cross-study data integration and longitudinal tracking. Note: COG IDs are not available for query and are never returned in response to a query. API, application programming interface; COG, Children's Oncology Group; CPI, CCDI Participant Index.

The CPI leverages direct and transitive associations between known identifiers that represent the same participant. Data submitters submit mappings of two or more identifiers that correspond to the same participant, along with information about the domain or namespace from which each ID originates (Fig 2). They are responsible for ensuring the accuracy and completeness of these mappings.

The process described above can be considered direct mappings since they come straight from the source (data submitters). In addition, the CPI has a process to identify mappings across domains indirectly (transitive associations). For example, Institute X submits a mapping between its internal IDs and a USI (PT1234 ßà PBABCD). Institute Y submits a mapping between its internal IDs and a USI (S98765 ßà PBABCD). The CPI database checks incoming mappings using a matching algorithm. If a domain/ID pair (USI_PBABCD) is identified across mappings, then the IDs that domain/ID pairs are mapped to are also mapped to each other (PT1234 ßà S98765; Fig 2).

The CPI database checks all incoming mappings submitted by data providers using domains (namespaces) categorized as follows:

• Data set: Refers to a single data set from a study where participant records are stored, typically represented by a dbGaP phs accession number (eg, *phs000720*, Genomic Sequencing of Pediatric Rhabdomyosarcoma).
• Study: Represents a programmatic reference to research initiatives such as TARGET or institution-specific equivalents of data set domains (eg, *sd_aq9kvn5p*, equivalent to *phs002276*).
• Organizational identifier: Denotes a project- or network-level namespace of unique participant identifiers (eg, dbGaP's SSTR, AACR-GENIE, Kids First, PCDC, St Jude Cloud, UCSC Treehouse, COG USI).

Note: Data set mappings reference physical data sets, whereas study and organizational identifier mappings reference projects, networks, or initiatives.

### ID Querying and Retrieval

CPI's primary function is to respond to mapping queries from cancer researchers—it does not return participant-level data. Instead, it confirms when the same participant appears in different CCDI data sets. To preserve data privacy and maintain the honest broker model, the CPI will never return COG IDs during any query (Fig 2). These identifiers are used only as anchor points during registration to cross-reference COG USIs and other shareable participant research IDs.

**FIG 3.**
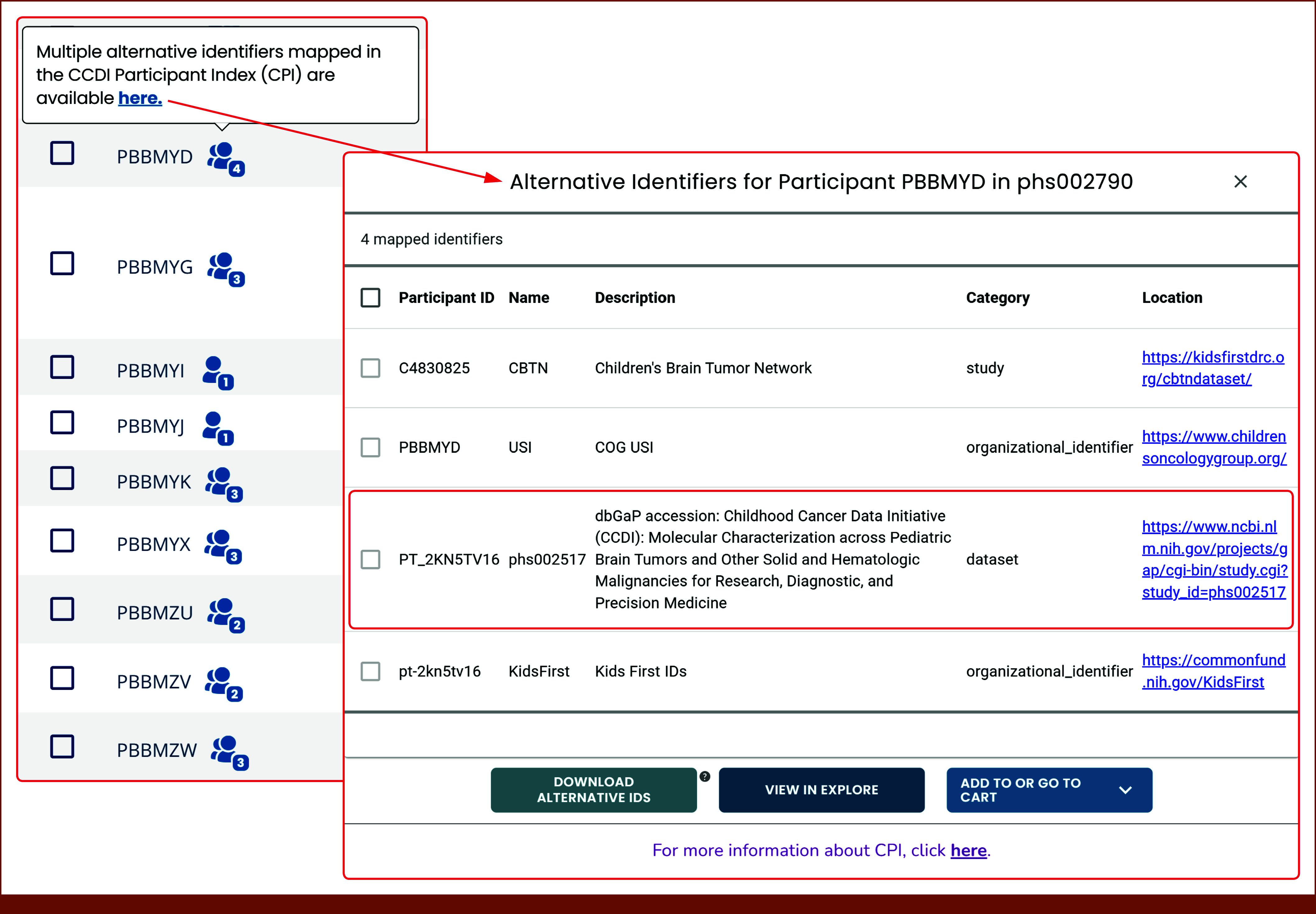
Participant tab view in the CCDI Hub Explore Dashboard displaying mapped identifiers. This screenshot shows how users can view mapped participant identifiers. From the Participants tab in the Explore Dashboard, users can select a participant ID to view its associated mappings across multiple data sets, studies, and contributing organizations. The CPI mappings window displays each record's name, description, category, and source location. Public participant ID mappings for a given study can also be found in the “Synonyms” tab of the downloadable manifest, available under the “Studies” tab in the CCDI Hub Explore Dashboard. CCDI, Childhood Cancer Data Initiative; CPI, CCDI Participant Index.

The CPI is not directly accessible to users; instead, it operates as a backend reference service for applications, systems, and services within the CCDE. For example, when a query is initiated through the CCDI Hub Explore Dashboard, the system retrieves matching participant research IDs. Through the Participant tab, participants with mappings in the CPI are displayed as an icon. These mappings are included as synonyms in manifests displayed under the Study view (Fig 3). The NCCR Data Platform serves as the primary source for building participant-level cohorts and identifying mappings to other CCDI and external resources.

The CPI API functions as a reference service that allows software applications to communicate with each other, providing information on participants' research IDs to authorized applications. Applications authorized by NCI can input participant IDs and retrieve all associated IDs along with their respective domains. This helps in mapping participants across different data sets and studies. Authorized users can access information about each domain within the CPI database, including the domain name, description, category, and a URL to additional data. Also available are participant statistics for each domain, including the number of participants mapped to various domains, along with the total number of participants. System owners interested in access may submit a request by e-mailing NCIChildhoodCancerDataInitiative@mail.nih.gov.

### CPI Statistics

New CCDI data submitters are encouraged to cross-reference their participant research IDs with other known identifiers whenever available. Data mappings are being incorporated from data sets (eg, dbGaP, St Jude, AACR GENIE, Kids First, COG) where participant-level ID matches have already undergone curation. Up-to-date lists of CPI domains and participant counts are available in the CPI Release Notes.^22^ As of October 2025, CPI has 63,875 of 443,476 total participants with mappings between two or more domains. Of these, there are 1,652 participants mapped between two dbGaP data sets and 11 participants mapped across three dbGaP data sets.

### Security, Privacy, and Compliance Safeguards

The CPI operates within NCI's Amazon Web Service hosting environment under a Federal Information Security Modernization Act/Federal Risk and Authorization Management Program Moderate Authority to Operate.^23^ Access is available to authorized users and systems that integrate with the CPI via API. As required under any federal system security plan, the CPI will log all queries and responses. Such logs are subject to audit, especially in the case of anomalous activity. The logs will also be used to collect usage metrics.

The CPI database stores only participant identifiers and does not store, transmit, or receive PII. It does not issue or distribute universal identifiers. While linking data sets enhances their scientific value and supports answering more complex research questions, mapping records at the participant level can also increase the potential risk of reidentification, even when data are fully deidentified. To mitigate these risks, the CPI does not store underlying primary data; instead, data access is governed by the policies and authorization mechanisms of the primary repositories that house those data. All data sets stored in National Institutes of Health (NIH) and NCI repositories comply with applicable NIH data access policies to ensure the responsible and secure use of participant-level data.

### Considerations for Data Interpretation and Cross-Analysis

The database model used by the CPI enables tracking of data without requiring recreation of mapping information for each use. While merging and deduplication are not performed centrally, the information needed to support cross-analysis is made available to researchers. Each data set is treated separately, so researchers should

• Make informed decisions about potentially conflicting data from multiple sources.
• Track new data added for a given participant over time and identify which studies include long-term follow-up information.
• Properly attribute data to the originating study or source.
• Adhere to any data use limitations associated with each data set (eg, a dbGaP data set with a disease-specific limitation is more constrained than the one approved for general research use, even if the same participant appears in both).

## DISCUSSION

Participant and sample identifiers play an essential role in accurately mapping and linking clinical, phenotypic, genomic, and other data types across the CCDE. Participants in research protocols and clinical trials often receive separate IDs when enrolled in different studies, leading to unintended data fragmentation that hinders the integration of clinical and research information. CCDI has observed that even within the same institution, different laboratories and research programs often assign separate participant IDs, creating challenges for integrating data across studies.

For prospective studies like MCI, CCDI proactively collects and integrates multimodal data originating from multiple sources: biospecimens and histopathology images from the Nationwide Children's Hospital (NCH) Biopathology Center; molecular characterization results from the NCH Institute for Genomic Medicine; and demographic, diagnostic, and follow-up data from COG using the COG USI to unify information across data sets. Longitudinal follow-up of participants with genetic abnormalities, as well as cascade testing of family members, will also be collected using USIs. For additional data types, such as EHR, treatment, and radiology imaging data collected at individual COG sites, the CPI can enable linkages as it maintains mappings between COG IDs and USIs, allowing sites that use only COG IDs to participate in integrated analyses.

For retrospective data sets, linkage is particularly challenging without identifiable information such as names or dates of birth, which cannot be shared across institutions. The CPI addresses this challenge by functioning as a mapping service that collects all known participant identifiers provided by data submitters to facilitate cross-referencing across data sets. When a participant has data associated with multiple research IDs from different studies or institutions, submitters are asked to include any other known identifiers referring to the same individual, to the extent of their knowledge. These cross-referential identifiers are then used to populate the CPI, improving linkage of retrospective data.

To promote data consistency and interoperability, CCDI encourages institutions to adopt privacy-preserving record linkage (PPRL) technologies for all future studies and protocols. Implementing PPRL approaches assigns Global Unique Identifiers (GUIDs),^24^ which enable secure linkage of participant records without exposing PII, ensuring that data generated across laboratories, studies, and systems can be connected accurately and safely. GUIDs play a pivotal role in enabling secondary data use by allowing researchers to conduct deeper analyses beyond the scope of the original research project. Some organizations like the American Medical Association^25^ have proposed creating a National Cancer Research Patient Identifier using PPRL technology. These types of unique IDs can be added to the CPI without modification as they become available.

In summary, the CPI functions as an online central registry that cross-references participant research identifiers across data sources to ensure consistency in data collection and promote interoperability and data integration. CCDI looks forward to continued collaboration with the research community to expand data contributions, strengthen interoperability, and build a unified, privacy-preserving resource that maximizes the potential impact and utility of pediatric cancer data for discovery and innovation.

## References

[1] Childhood Cancer Data Initiative (CCDI). National Cancer Institute. https://www.cancer.gov/research/areas/childhood/childhood-cancer-data-initiative

[2] About the Childhood Cancer Data Initiative Data Ecosystem. National Cancer Institute. https://www.cancer.gov/research/areas/childhood/childhood-cancer-data-initiative/data-ecosystem

[3] Jagu S, Mardis ER, Wedekind MF, et al: Childhood Cancer Data Initiative: Status report. Pediatr Blood Cancer 71:e30745, 2024 37889049 10.1002/pbc.30745

[4] Flores-Toro JA, Jagu S, Armstrong GT, et al: The Childhood Cancer Data Initiative: Using the power of data to learn from and improve outcomes for every child and young adult with pediatric cancer. J Clin Oncol 41:4045-4053, 2023 37267580 10.1200/JCO.22.02208 PMC10461939

[5] Jagu S, Guidry Auvil JM, Reaman G: The childhood cancer data initiative: Enabling data sharing to drive research advances and transform pediatric cancer diagnosis and treatment. Curr Opin Pediatr 37:42-47, 2025 39699099 10.1097/MOP.0000000000001422 PMC11658014

[6] Flores-Toro J, Jagu S, Smith M, et al: Childhood Cancer Data Initiative: Expanded access to tumor molecular profiling for children, adolescents, and young adults. J Natl Cancer Inst 118:199-204, 2026 40794906 10.1093/jnci/djaf214 PMC12360469

[7] Volchenboum SL, Cox SM, Heath A, et al: Data commons to support pediatric cancer research. Am Soc Clin Oncol Educ Book 37:746-752, 2017 28561664 10.1200/EDBK_175029

[8] Tryka KA, Hao L, Sturcke A, et al: NCBI's database of genotypes and phenotypes: dbGaP. Nucleic Acids Res 42:D975-D979, 2014 24297256 10.1093/nar/gkt1211 PMC3965052

[9] AACR Project GENIE Consortium: AACR Project GENIE: Powering precision medicine through an international consortium. Cancer Discov 7:818-831, 2017 28572459 10.1158/2159-8290.CD-17-0151 PMC5611790

[10] Withycombe JS, Alonzo TA, Wilkins-Sanchez MA, et al: The Children's Oncology Group: Organizational structure, membership, and institutional characteristics. J Pediatr Oncol Nurs 36:24-34, 2019 30426816 10.1177/1043454218810141 PMC6389409

[11] Plana A, Furner B, Palese M, et al: Pediatric cancer data commons: Federating and democratizing data for childhood cancer research. JCO Clin Cancer Inform 10.1200/CCI.21.00075 34662145

[12] Jagu S, Guidry Auvil JM, Cunningham M, et al: Building pediatric cancer cohorts and accessing data using Childhood Cancer Data Initiative tools. JCO Clin Cancer Inform 10.1200/CCI-25-00217 PMC12724070 41370779

[13] Lupo PJ, Siegel DA, Schussler NC, et al: Enrollment in Children's Oncology Group's clinical trials: Population-based linkage with the National Childhood Cancer Registry. J Natl Cancer Inst 117:1868-1874, 2025 40515409 10.1093/jnci/djaf134 PMC12415956

[14] About Us. National Cancer Institute NCTN/NCORP Data Archive. https://nctn-data-archive.nci.nih.gov/about-us

[15] Jagu S, Smith MA, Seibel NL, et al: The impact of focused federal initiatives on pediatric cancer research. Cancer 131:e70172, 2025 41237091 10.1002/cncr.70172 PMC12617966

[16] Explore: National Cancer Institute Childhood Cancer Data Initiative Hub. https://ccdi.cancer.gov/explore

[17] CCDI Hub Data Federation Resource. National Cancer Institute Childhood Cancer Data Initiative Hub. https://ccdi.cancer.gov/data-federation-resource

[18] Hudson A, Fournier M, Coulombe J, et al: Using existing pediatric cancer data from the Gabriella Miller Kids First Data Resource Program. JNCI Cancer Spectr 7:pkad079, 2023 37788089 10.1093/jncics/pkad079 PMC10635640

[19] McLeod C, Gout AM, Zhou X, et al: St. Jude Cloud: A pediatric cancer genomic data-sharing ecosystem. Cancer Discov 11:1082-1099, 2021 33408242 10.1158/2159-8290.CD-20-1230 PMC8102307

[20] Treehouse Childhood Cancer Initiative: UC Santa Cruz Genomics Institute. https://treehousegenomics.ucsc.edu/

[21] Chapman OS, Sridhar S, Chow EY, et al: Extrachromosomal DNA associates with poor survival across a broad spectrum of childhood solid tumors. medRxiv. 10.1101/2025.07.22.24308163

[22] CCDI Participant Index (V1.2.0). https://participantindex-docs.ccdi.cancer.gov/

[23] Federal Information Security Modernization Act FISMA: NIST Information Technology Laboratory Computer Security Resource Center. https://csrc.nist.gov/topics/laws-and-regulations/laws/FISMA

[24] McMurry JA, Juty N, Blomberg N, et al: Identifiers for the 21st century: How to design, provision, and reuse persistent identifiers to maximize utility and impact of life science data. PLoS Biol 15:e2001414, 2017 28662064 10.1371/journal.pbio.2001414 PMC5490878

[25] American Medical Association House of Delegates. https://www.ama-assn.org/system/files/a22-021.pdf

